# Hybrid Surface Acoustic Wave- Electrohydrodynamic Atomization (SAW-EHDA) For the Development of Functional Thin Films

**DOI:** 10.1038/srep15178

**Published:** 2015-10-19

**Authors:** Kyung Hyun Choi, Hyun Bum Kim, Kamran Ali, Memoon Sajid, Ghayas Uddin Siddiqui, Dong Eui Chang, Hyung Chan Kim, Jeong Beom Ko, Hyun Woo Dang, Yang Hoi Doh

**Affiliations:** 1 Department of Mechatronics Engineering, Jeju National University, Jeju 690-756, South Korea; 2 Department of Applied Mathematics University of Waterloo, Canada; 3 Korea Institute of Industrial Technology, South Korea; 4 Department of Electronics Engineering, Jeju National University, Jeju 690-756, South Korea

## Abstract

Conventional surface acoustic wave - electrostatic deposition (SAW-ED) technology is struggling to compete with other thin film fabrication technologies because of its limitation in atomizing high density solutions or solutions with strong inter-particle bonding that requires very high frequency (100 MHz) and power. In this study, a hybrid surface acoustic wave - electrohydrodynamic atomization (SAW-EHDA) system has been introduced to overcome this problem by integrating EHDA with SAW to achieve the deposition of different types of conductive inks at lower frequency (19.8 MHZ) and power. Three materials, Poly [2-methoxy-5-(2-ethylhexyloxy)-1, 4-phenylenevinylene] (MEH-PPV), Zinc Oxide (ZnO), and Poly(3, 4-ethylenedioxythiophene):Polystyrene Sulfonate (PEDOT:PSS) have been successfully deposited as thin films through the hybrid SAW-EHDA. The films showed good morphological, chemical, electrical, and optical characteristics. To further evaluate the characteristics of deposited films, a humidity sensor was fabricated with active layer of PEDOT:PSS deposited using the SAW-EHDA system. The response of sensor was outstanding and much better when compared to similar sensors fabricated using other manufacturing techniques. The results of the device and the films’ characteristics suggest that the hybrid SAW-EHDA technology has high potential to efficiently produce wide variety of thin films and thus predict its promising future in certain areas of printed electronics.

The science and technology of thin films has been playing a major role in high tech industries. The thin film industry has been present for the last few decades, and because of its immense importance and unique capabilities, the application areas of thin films have been widely expanded. The most notable of these are microelectronic devices, optical coatings, biological implants, wear resistant coatings, flat panel displays, photovoltaic cells, and sensors^1^,^2^,^3^,^4^,^5^. A large variety of materials including metals, pure elements, organic materials, and compounds such as oxides, nitrides, and polymers can be deposited on different types of substrates such as metals, ceramics, and polymers, through thin film technologies^6^,^7^,^8^. This is because of the continuously changing needs and requirements of various science fields, whereby thin film technologies have been continuously developed and more sophisticated and advanced trends have been introduced to fulfill the needs of the growing industry. Some of the most widely used thin film technologies are spin coating, chemical vapor deposition, atomic layer deposition, sputtering, spin coater, electro- hydrodynamic atomization, micro gravure printing, roll-to-roll atmospheric atomic layer deposition, and screen printing^9^,^10^,^11^,^12^.

One of the possible candidates for thin film fabrication and pattern deposition is the technology of surface acoustic wave (SAW) atomization^13^. SAW waves are very high frequency surface Raleigh waves travelling in the piezoelectric substrate and can energize droplet particles through vibrations, resulting in atomization and producing a dry fog type mist of droplets^14^,^15^,^16^. Realizing the fine atomization of the conductive inks through SAW, *Kim et al.* applied it in the fabrication of protein chips by incorporating SAW with electronic deposition (SAW-ED), where the charged atomized particles of the conductive proteins were moved opposite to gravity using a strong electric field^17^. This led to the introduction of a new method that could be used for patterns and film deposition. While the collection efficiency was improved by using a charge collimator, the deposition was not continuous as the droplet was placed at the interdigital transducer (IDT) repeatedly after atomization. Also, due to the large active volume of the droplet, the atomized particle size was slightly less than 10 μm, which while being no smaller than the minimum achievable droplet size of EHDA, only avoids the problems of nozzle blocking. The issue was addressed and the SAW frequency was increased to up to 95 MHz to produce sub-micron particles^18^. Currently, the problems with SAW-ED include fabrication of an IDT capable of operating at such high frequency is very expensive, and also, the atomization is not continuous, affecting the deposition time, efficiency, and quality^19^,^20^. Protein particles of diameter 1–10 μm have been produced for aerosols and drug delivery using high frequency SAW with the same shortcomings as mentioned above. Furthermore, in most of the work on SAW atomizers, only highly conductive low density proteins are used due to their ease of atomization and deposition. In 2011, filter paper and a syringe reservoir were used for a continuous supply of ink, utilizing the “self-pumping effect” of SAW for the continuous atomization of the liquids^21^. Unfortunately, this method is only useful for low molecular weight liquids and thus greatly limits the ink selection choice for device fabrication. In the same year, an attempt was made for thin film and pattern deposition of conductive ink using SAW-EHDA by providing continuous ink flow through a syringe pump, but the film characteristics were very poor^22^. It could thus be assumed that we need a hybrid SAW-EHDA when the EHDA also works on electric atomization of the fluid and can produce very uniform thin films. SAW-EHDA is a strong candidate for thin film fabrication compared to EHDA because the atomization is very uniform and the particle size achieved through SAW atomizers is very small (sub-micron) compared to EHDA systems (few microns). As the particle size becomes smaller, the minimum achievable film thickness also reduces, while the film will be more uniform and non-porous with better electrical and mechanical properties.

In this research work, the limitations related to SAW and EHDA have been addressed and overcome through unique hybrid atomization and deposition technology. The benefits of SAW and EHDA are combined and the drawbacks are eliminated by using a dual atomization setup. EHDA is used as the first atomizer that continuously supplies small droplets of inks to the IDT of the SAW atomizer and the droplets are further broken down into sub-micron size by SAW waves. This method decreases the active volume of the liquid to be atomized using SAW, thus reducing the required frequency to achieve smaller droplets. Also, it ensures very uniform and continuous ink supply to the SAW atomizer. Another major advantage of this is that we can use a variety of different materials as the introduction of a pre-atomizer in the form of EHDA widens the density and molecular weight ranges. The comparison of SAW-EHDA with other deposition techniques in terms of deposition rate (thickness), electrical results, transparency and surface roughness for MEH-PPV, PEDOT:PSS and ZnO films has been represented in supplementary Table 1. We hope that this method will be the next generation high potential technology for the fabrication of functional thin films and patterns.

## Results

### Surface Acoustic Wave Electrohydrodynamic Hybrid Atomization for Film Deposition

Surface Acoustic Wave (SAW) combined with Electrohydrodynamic Atomization (EHDA) is a method of electronic deposition of conductive materials either in the form of thin films or developing patterns using masks. This hybrid system incorporates both SAW and EHDA together for the first time for thin films and pattern fabrication. In the SAW based system, an Inter Digitated Transducer (IDT) was used to generate high frequency surface Rayleigh waves in a piezoelectric substrate (Y-LiNbO_3_). Rayleigh waves are surface acoustic waves that are generated when the anisotropic Y-LiNbO_3_ is rotated 128°^22^. The Rayleigh wave displacement equations solved by *Choi et al.* show that the elliptical rotation is in the opposite direction of wave propagation^22^. The maximum frequency of the waves depends on the properties of the IDT substrate material and the dimensions of IDT. In this case, the frequency supplied to the IDT was 19.8 MHz. To achieve this frequency, the dimensions of the IDT were to be calculated using equation 1.


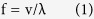


Here, “f” is the frequency that is 19.8 MHz, “v” is the acoustic velocity of the Rayleigh wave for LiNbO_3_ piezoelectric that is known to be 3960 m/s, and “λ” is the wavelength of the travelling wave that dictates the dimensions of the IDT, depending on the other two parameters, or vice versa. “λ” must be equal to the pitch of the IDT, which means that the electrode width and the gap between the two electrodes should be equal to “λ/4” i.e., 50 μm each^23^. The dimensions of the IDT used in this study are shown in supplementary Figure 1.

### Electrohydrodynamic Atomization

Electrohydrodynamic atomization (EHDA) has been thoroughly studied and different relations of particle size and other parameters have been developed. The three major issues involved in this process are: (i) Formation of an electrically driven jet. (ii) Deployment of the jet to a substrate, and (iii) Ordering of particles in the printed features^24^. The first step is the formation of a stable cone jet of which, the diameter is mainly dependent upon the liquid flow rate and conductivity. The parameters influencing the droplet production process are conductivity, dielectric permittivity, surface tension, liquid flow rate, density, viscosity, applied potential, capillary shape, and material wettability^25^. The phases involved in the formation of a stable cone jet are: (i) Pendant Drop (ii) Cone (iii) Cone Jet, and (iv) Stable Cone jet^26^. The average critical voltage that supports the meniscus on the capillary tube was calculated to be ~1 KV. The droplet size depends on the density (*ρ*) of the liquid, ink flow rate (Q), surface tension (*γ*), and ink conductivity (K). Droplets ranging within a few microns diameter are achieved through EHDA.

### The Hybrid Surface Acoustic Wave- Electrohydrodynamic Atomization (SAW-EHDA) System

In previous studies regarding SAW-EHDA, the ink is incident on the SAW IDT device drop by drop through a syringe to obtain a continuous flow and deposition. The problem with this type of setup was that when using a high density solution or a solution with strong inter-particle bonding, the drop of the ink remained at the IDT and SAW waves did not have sufficient energy to break it into particles. The control volume on the substrate is the amount of liquid present at a time that can be atomized into particles and is dependent on the energy density of the acoustic wave. This energy density is the sum of the kinetic energy and the internal energy of the SAW substrate through which the waves are travelling. This energy is represented by equation 6^27^.


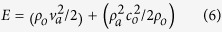


Here, *ρ*_*0*_ is the density change of the medium, *ρ*_*a*_ is the density change of the particles, *v*_*a*_ is the speed of a particle, and *c*_*o*_ is the sound speed. The emission pressure on the control volume is the time average of the energy. As the control volume decreases, the emission pressure on the particles will be more effective and the force required to break the attraction between the molecules of the solution for atomization will decrease. Also, the density change in the medium is due to the SAW waves travelling through the piezo-electric substrate and the density change of particles of the material present on top of the IDT in the control volume plays a vital role in the determination of the internal energy and the atomization efficiency. The density of the particles is significantly altered in case of Electrohydrodynamic atomization with a mist of micron level droplets as the ink supply to the IDT, as compared to that in case of the ink supply in the form of big drops.

**Figure 1:**
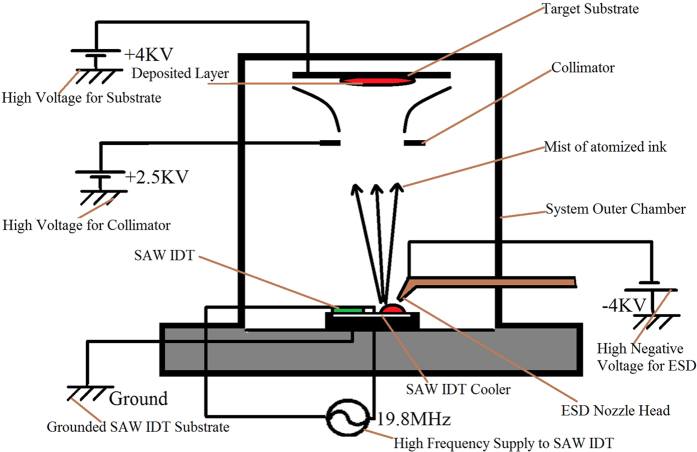
System details and working principle of the Hybrid SAW-EHDA deposition system.

In previous studies, where the continuous flow of ink over the substrate was achieved by dropping the ink on the surface of the substrate, the atomization needed to be conducted at a very high frequency, with greater input power, and a sufficiently strong emission pressure and force to break the bonding of a large volume of material. This demands that the IDT design is capable of handling the high frequency vibrations of up to 100 MHz. The overall system cost is increased and the energy requirements are elevated. This problem has been overcome by the hybrid system proposed and tested by integrating EHDA with SAW to achieve the deposition of different types of conductive inks at a low frequency and a lower input power. The control volume is largely reduced, decreasing the required force for atomization. This method allowed us to deposit metallic conductive inks with high density using SAW-EHDA, and other conductive and semi-conductive polymers were also deposited. The integration of EHDA with SAW also sufficiently increases the deposition speed of the SAW atomization process and thus reduces the overall deposition time. The system details and working principle is shown in Fig. 1.

The material is first sprayed through EHDA onto the SAW-IDT and the elastic surface Raleigh waves then further atomized the droplets, producing a fine mist of charged particles that are attracted to the deposition substrate through an electric field applied between the mask and IDT. Particles in the range of a few microns were sprayed on the surface of SAW IDT and were further atomized into particles of very small size in a submicron range as shown in supplementary Figure 3. These charged particles were attracted using an electric field towards the target substrate. The electric field was applied using a four point voltage system. A high negative voltage in magnitudes of kilo Volts was applied across the EHDA nozzle and a ground terminal was attached to the surface at which the SAW IDT was placed. The ground attracted the negatively charged particles and made them incident directly on the IDT. The particles further atomized by the SAW phenomenon were attracted towards the target substrate through a positive electric field applied by supplying a high magnitude positive voltage at the target substrate (Video S1 and S2 in Supplementary Information). A charge collimator was used to enhance the particle collection at the substrate. The collimator was supplied with a slightly lower positive voltage than that of the target substrate in kilo Volts and was positioned between the SAW IDT and the target substrate. Electric field simulations were performed to maximize the particle collection at the target substrate that is also a major factor in defining the efficiency of the SAW-EHDA process. The simulations presented in supplementary Figure 2 show that the efficiency of the electric force is increased from 10% to 33% with the introduction of a collimator at the collector end^22^.

### Experimental Setup

The overall experimental setup for the hybrid SAW-EHDA system developed for thin film deposition and patterning is shown in Fig. 1. The system consists of a syringe pump system that pumps the ink under experimentation to the ESD spray head at a continuous flow rate and pressure. The spray head is a metallic nozzle with a diameter of hundreds of microns. The nozzle is supplied with a high voltage negative supply of several kilo Volts magnitude. The spray is targeted on the SAW IDT, which is placed on a grounded metallic substrate cooled by circulating water and a thermo-electric Peltier cooler chip. The target substrate is on the top of the system which is attached to a 3-axis movable stage along with the collimator controlled using a labview program. Both the target substrate and the collimator are supplied with different positive voltages in several kilo Volts magnitudes. The voltage of the collimator was less than that of the target substrate to produce an electric field between them and to allow the particles to move through them to the final target. The distance between the EHDA nozzle and the SAW substrate was kept fixed at 5 mm, while the distance between the collimator and the EHDA nozzle was varied according to the ink under experimentation. The distances were optimized so that the conductive ink would first drop from the EHDA nozzle to the SAW device through a high voltage potential difference, then attract towards the collimator, and finally to the destination target substrate through an opposite electric applied field.

### Thin film fabrication through SAW-EHDA

**Figure 2:**
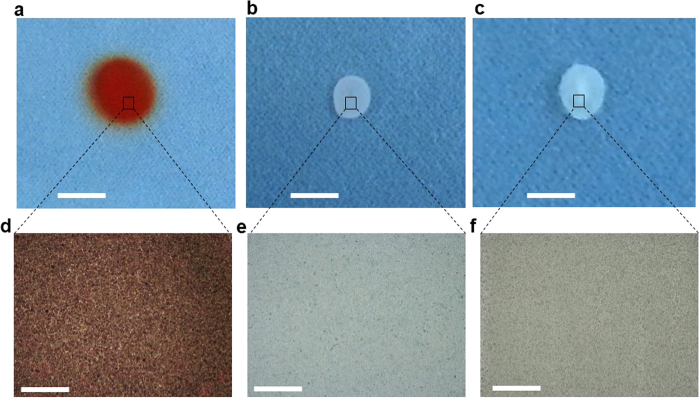
Low and high magnification optical micrographs for the SAW-EHDA developed MEH-PPV, PEDOT:PSS, and ZnO thin films. (**a**) MEH-PPV thin films fabricated using a mask with a size of 5 mm. (**b**) PEDOT:PSS thin films fabricated using a mask with a size of 3 mm. (**c**) ZnO thin films fabricated using a mask with a size of 3 mm. (**d**) High magnification image of MEH-PPV thin films showing no irregularities such as cracks and pin holes. (**e**) High magnification image of PEDOT:PSS thin films showing no irregularities. (**f**) High magnification image of ZnO thin films showing no irregularities such as cracks and pin holes. The scale bar size for Fig. a, b, and c is 5 mm and that for d, e, and f is 10 um.

The fabrication of MEH-PPV, PEDOT:PSS, and ZnO thin films through SAW-EHDA was performed under a set of carefully optimized processing parameters, which are presented in Table 1. It is evident that most of the experimental parameters that have been used for the deposition of different functional thin films are quite similar. The intention behind the implemented approach of utilizing the same parameters, as much possible, was to effectively investigate and better understand the true potential of this unique technology to develop a wide variety of thin films. It would have been impossible to compare the results of MEH-PPV, PEDOT:PSS, and ZnO thin films if the processing parameters such as nozzle diameter, flow rate, and deposition time, etc. were of different values. One of the main parameters, applied voltage, is mainly dependent on the properties of the deposited material. Therefore, its values have differed for MEH-PPV, PEDOT:PSS, and ZnO, even though most of the other experimental parameters have been kept the same. It is worth mentioning here that the values of the applied voltage and power that have been utilized to deposit ZnO (3.52 kV, 6 W) are higher than those of MEH-PPV (3.27 kV, 5 W) and PEDOT:PSS (3.35 kV and 5 W). This is because ZnO is a comparatively heavier material than either MEH-PPV or PEDOT:PSS, since the density of ZnO is 5.16 g/cm^3^ and that of MEH-PPV and PEDOT:PSS is 0.8 g/cm^3^ and 1 g/cm^3^, respectively. Therefore, ZnO requires a higher voltage and power so that it can be vaporized and transported to the substrate. The optical micrographs of the SAW-EHDA fabricated MEH-PPV, PEDOT:PSS, and ZnO thin films under the optimized set of parameters have been shown in Fig. 2.

**Figure 3:**
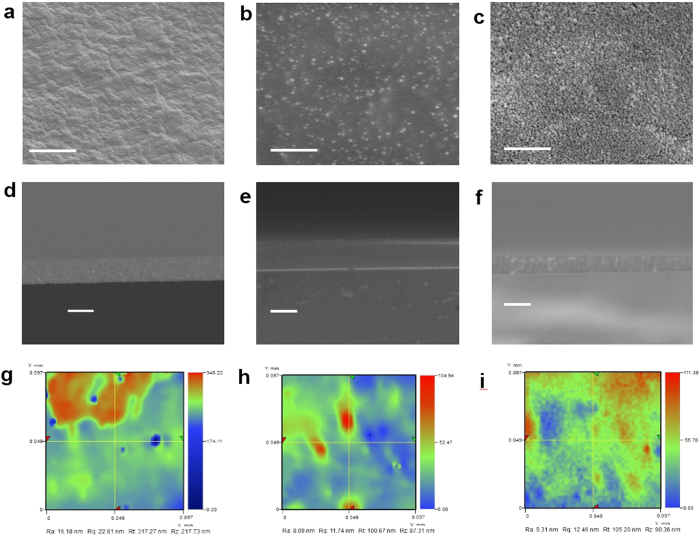
FESEM images for surface and cross sectional analysis and 2D surface profiles for the quantitative analysis of the SAW-EHDA developed MEH-PPV, PEDOT:PSS, and ZnO thin films. (**a**) MEH-PPV thin films showing no cracks or pin holes, but showing a rough morphology. (**b**) PEDOT:PSS thin films with no irregularities and a smooth morphology. (**c**) ZnO thin films with no irregularities and smooth morphology. (**d**) FESEM cross sectional image for MEH-PPV thin films showing thickness of 2.035 um. (**e**) PEDOT:PSS cross sectional image showing thickness of 2.120 um. (**f**) ZnO cross sectional image showing thickness of 1.625 um. (**g**) 2D surface profile of MEH-PPV thin films showing average arithmetic roughness (Ra) of 16.18 nm. (**h**) 2D surface profile of PEDOT:PSS thin films showing average arithmetic roughness (Ra) of 8.09 nm. (**i**) 2D surface profile of ZnO thin films showing average arithmetic roughness (Ra) of 9.31 nm. The scale bar size for Fig. a, b, and c is 200 nm and that for d, e, and f is 2 um.

Figure 2a–c show the low resolution images of the MEH-PPV, PEDOT:PSS, and ZnO thin films, respectively. The high resolution micrographs of the respective thin films are also shown in Fig. 2d–f. The surface morphology of the developed thin films was carefully analyzed through field emission scanning electron microscopy (FESEM). Apart from the thickness measurement using the state-of-the-art nondestructive thin film thickness measurement system, the thickness was also confirmed through cross sectional analysis of thin films using FESEM. In order to obtain quantitative results regarding the quality of the surface morphology, all three types of films were carefully characterized using a 3D nano non-contact surface profiler. The FESEM images of surface morphology and thickness as well as the 2D surface profile images of MEH-PPV, PEDOT:PSS, and ZnO thin films are shown in Fig. 3.

**Figure 4:**
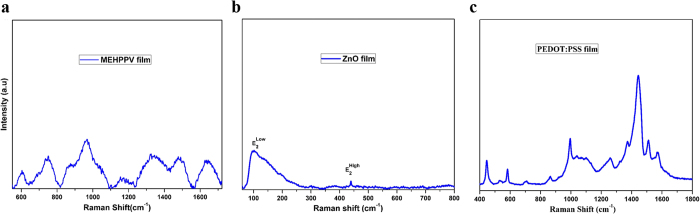
Raman spectroscopy of as deposited films through SAW-EHDA. (**a**) Raman scattering of MEH-PPV film (**b**) ZnO film (**c**) PEDOT:PSS film.

**Figure 5:**
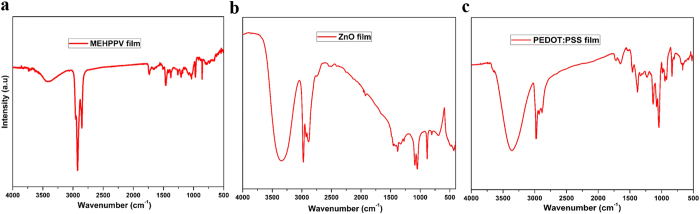
FT-IR spectroscopy of as deposited films through SAW-EHDA. (**a**) FT-IR spectrum of MEH-PPV film (**b**) ZnO film (**c**) PEDOT:PSS film.

Raman spectroscopy is a powerful characterization tool in the study of thin films, whereas Fourier Transform-Infrared (FT-IR) spectra help in identifying the presence of functional groups. Chemical and structural investigation of the MEH-PPV, ZnO, and PEDOT:PSS films deposited through SAW-EHDA were carried out using Raman scattering and FT-IR spectroscopy as shown in Figs 4 and 5, respectively. Figure 4a–c indicate the Raman spectra of the films, while Fig. 5a–c show the FT-IR spectra of the deposited films.

**Figure 6:**
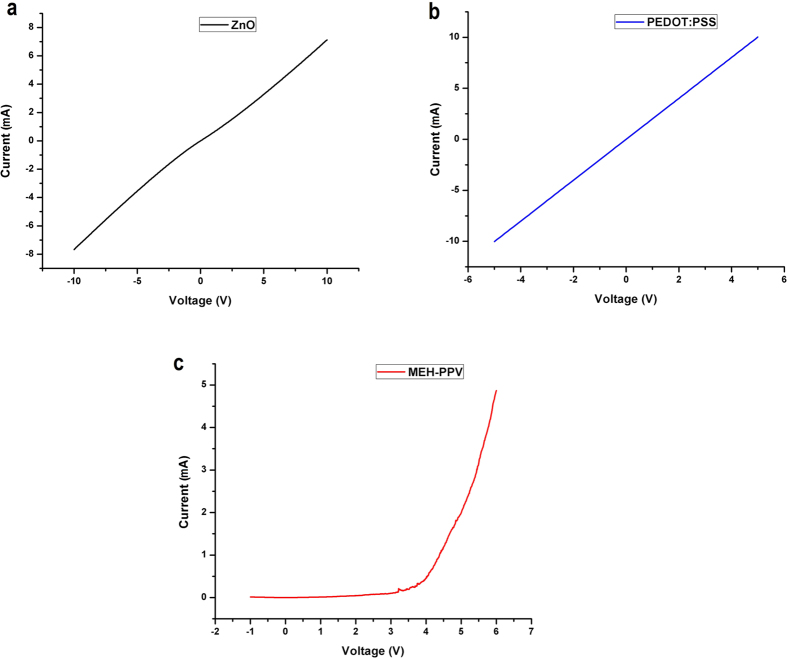
I–V curves of the deposited conductive thin films through the hybrid SAW-EHDA process (**a**) I–V Curve of ZnO thin film showing Ohmic behavior of the film, (**b**) PEDOT:PSS thin film I–V curve showing Ohmic behavior of the film, (**c**) MEH-PPV thin film I–V curve showing semi-conductive diodic behavior with a turn on voltage of 4V.

Electrical characterization of the fabricated conductive thin films was carried out to check whether the film quality and conductivity were comparable with the conventional fabrication methods. For electrical characterization, four circular silver contacts each with a diameter of 0.5 mm at a separation distance of 3 mm from each other forming a square were deposited on top of the surfaces. Then the I-V data was recorded by connecting the probes of the device analyzer to any two of the contacts at a time. All the four I–V curves were almost identical and one of them for each of MEH-PPV, PEDOT:PSS, and ZnO deposited through the hybrid SAW-EHDA process are presented in Fig. 6. From Fig. 6a, the resistance of ZnO can be calculated as 1.303 KΩ while using Fig. 6b, the resistance of PEDOT:PSS comes out to be around 498 Ω. MEH-PPV shows semi-conductive behavior, as shown in Fig. 6c, with a turn on voltage of around 4 V with 0.6 mA current flow at the turn on voltage while a current of 4.9 mA at 6 V showing a resistance of 1.22 KΩ. The measured thicknesses of the MEH-PPV, PEDOT:PSS, and ZnO thin films are 2.035 μm, 2. 120 μm, and 1.625 μm, respectively as determined from the cross-sectional SEM images of Fig. 3. The sheet resistance of the films can be calculated by using the Van der Pauw method. In our case, all the four resistance values were almost equal for one film, so, an approximation of R_ver_ = R_hor_ = R is taken for simpler calculations and the sheet resistance for every film is calculated using the formula R_s_ = πR/ln (2). This gives us the sheet resistance of ZnO thin film to be equal to 5.9 KΩ/ □, sheet resistance of PEDOT:PSS thin film to be 2.26 KΩ/□, and that of MEH-PPV to be 5.53 KΩ/□. The resistivity of the films can be calculated by multiplying the sheet resistance with the film thickness. The resistivity of ZnO film is equal to 959 mΩ-cm, for PEDOT:PSS it is 479 mΩ-cm, and for MEH-PPV it is 1.125 Ω-cm. The conductivity of the thin films can be calculated by taking inverse of the resistivity which gives us the conductivity for ZnO to be 1.04 S/cm while the conductivity of PEDOT:PSS film is calculated to be 2.09 S/cm, and 0.889 S/cm for MEH-PPV. All the deposited films show promising electrical characteristics that are comparable to the previously reported results of the respective films.

## Discussion

Analyzing the morphological features of thin films not only provides information regarding the feasibility, optimization, and degree of correctness in the operational parameters that have been used, but also allows to predict about the performance of such functional thin films. It is known that if the films contain irregularities such as cracks, pinholes, and scratches, they will be vulnerable to various elements such as atmospheric moisture and oxygen, which can penetrate through such irregularities and can deteriorate the performance of the films of the devices in which they are used. Therefore, it is very important to study the surface morphology thoroughly.

The optical micrographs presented in Fig. 2a–c show that MEH-PPV, PEDOT:PSS, and ZnO have been deposited quite uniformly over the substrate. The scale bar shows that the film area for MEH-PPV is larger than that of PEDOT: PSS and ZnO. This is because a larger size mask with a diameter of 5 mm was used for MEH-PPV deposition than the 3 mm mask used for PEDOT: PSS and ZnO. The high resolution micrographs clearly show that the developed films show no cracks or scratches. Although the micrographs concluded good morphology of the films, a much more intense and deeper study of the morphology is required, which has been performed through FESEM. As presented in Fig. 3a–c, the high resolution FESEM images of MEH-PPV, PEDOT:PSS, and ZnO thin films respectively indicate that they are free of cracks and pin holes. The absence of such irregularities confirms that the films are of good quality and can be used in printed electronic applications. However, the results show that the surface morphology of MEH-PPV films is comparatively rougher than that of the PEDOT: PSS and ZnO thin films. The study of thickness of the developed films provides information related to the growth and uniformity of the developed films. Fig. 3d–f show the cross sectional FESEM images of MEH-PPV, PEDOT: PSS, and ZnO thin films respectively. It is evident from the figures that the developed films are very smooth and highly uniform. The measured thickness of the MEH-PPV, PEDOT:PSS, and ZnO thin films are 2.035 μm, 2.120 μm, and 1.625 μm, respectively. As the deposition time was maintained i.e., 30 min for all three materials, the calculated deposition rates for MEH-PPV, PEDOT: PSS, and ZnO thin films were 0.067 μm/min, 0.070 μm/min, and 0.054 μm/min, respectively. It is evident from the results that the deposition rate of ZnO was comparatively lower than those of MEH-PPV and PEDOT: PSS. This is mainly because of the fact that ZnO is a comparatively heavier material than MEH-PPV and PEDOT:PSS because the density of ZnO is 5.16 g/cm^3^ and that of MEH-PPV and PEDOT:PSS is 0.8 g/cm^3^ and 1 g/cm^3^, a respectively. The thickness of the developed films was also measured with thickness measurement system K-MAC ST4000-DLX and the results were quite comparable to those calculated through cross sectional analysis of the films through FESEM.

The morphological results were further confirmed and examined through a nano surface profiler for the sake of quantitative analysis. Figure 3g–i show the 2D surface profiles of the SAW-EHDA developed MEH-PPV, PEDOT:PSS, and ZnO thin films, respectively. The results showed that the average arithmetic roughness (Ra) of MEH-PPV, PEDOT:PSS, and ZnO thin films was 16.18 nm, 8.09 nm, and 9.31 nm, respectively. As evident from the results, the developed MEH-PPV thin films are of comparatively high surface roughness to that of PEDOT:PSS, and ZnO, confirming the results achieved from FESEM analysis. The average arithmetic roughness is the arithmetic average height of peaks and valleys from the mean line, measured within the sample of specific length. It is calculated as an integral of the absolute value of the roughness profile height over the evaluation length. It gives an effective description of the height variations in the surface. Apart from the (Ra), the figure also features other surface roughness parameters such as Rq, Rt and Rz. The Rq the as root mean square roughness and is described as the average height deviation from the reference line/plane across the entire area. Rt is described as the maximum peak to valley height in the specific sampling length, whereas Rz is the average peak to valley roughness. The Rq, Rt and Rz values for the MEH-PPV thin films are 22. 81 nm, 317.27 nm, and 217.73 nm respectively. Whereas the Rq, Rt and Rz values for the PEDOT:PSS thin films are 11.74 nm, 100.67 nm, and 87.31 nm and that for ZnO are 12.46 nm, 105.20 nm, and 90.36 nm respectively. The surface roughness values of the developed thin films are highly appreciable and are quite comparable to previously reported results for thin films being developed through EHDA technology which is greatly used for multi-layer device fabrication^28^,^29^,^30^. The 3D surface profiles for MEH-PPV, PEDOT:PSS, and ZnO thin films have been shown in supplementary Figures 4,5, and 6 respectively. The developed films were also characterized for optical properties. The results showed higher optical transmittance of more than 85% in the visible region for ZnO and MEH-PPV films, whereas the transmittance of PEDOT:PSS thin film was lower than 85%. The optical absorbance of films was also analyzed and was normalized by the thickness of films. The absorbance spectra of the SAW-EHDA fabricated MEH-PPV, PEDOT: PSS, and ZnO thin films have been shown in supplementary Figure 7.

The characteristic bands of MEH-PPV film in Raman scattering indicate C−H out of plane bending mode at 970 cm^−1^, 1155 cm^−1^ (mixture of C−C stretching and C−H in-plane bending liberation), C = C bonds at 1325 cm^−1^ (C = C stretching band of benzene), and 1634 cm^−1^ (C = C stretching band of vinyl group). The Raman spectra of ZnO deposited film through SAW-EHDA have characteristic peaks, where two peaks are observed at 99.9 cm^−1^ and 439 cm^−1^, respectively. The prominent peak at 99.9 cm^−1^ corresponds to the 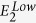 phonon mode of ZnO which is attributed to the lattice vibrations of zinc atoms. The 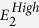 phonon mode being observed at 439 cm^−1^ is used to characterize the stress in the ZnO lattice. In bulk ZnO, this mode occurs at 434 cm^−1^ but in ZnO film it is shifted at 439 cm^−1^^31^. These characteristic vibrations are related to the lattice vibration of the oxygen atoms and indicate the wurtzite phase of ZnO. In the Raman spectra of the PEDOT:PSS film, the most prominent peak is observed between 1400 and 1500 cm^−1^. This narrow band is the characteristic of PEDOT:PSS film while in pristine PEDO:PSS this band is more broader^32^. Various bands are observed that are related to the polymer structure of PEDOT:PSS. The bands at 1568 cm^−1^ and 1511 cm^−1^ are attributed to the asymmetric C_α_ = C_β_ stretching, 1444 cm^−1^ is attributed to symmetric C_α_ = C_β_, 1373 cm^−1^ is attributed to C_β_−C_β_ stretching, and 1258 cm^−1^ is attributed to C_α_−C_α_ inter ring stretching. The band at 1040 cm^−1^ shows C−O−C deformation, 995 cm^−1^ is attributed to oxy-ethylene ring deformation, 708 cm^−1^ is attributed to symmetric C−S−C deformation, 582 cm^−1^ is attributed to oxy-ethylene deformation, and 446 cm^−1^ is attributed to SO_2_ bending.

The FT-IR spectra of deposited MEH-PPV film have various characteristics. Strong in-plane transitions are observed between 500 and 1500 cm^−1^, and most of the peaks correspond to the in-plane C−H bands. The medium strength experimental transition at 772 cm^−1^ can be interpreted to be an out of plane ring band of the phenylene rings. The peaks at 857 and 968 cm^−1^ correspond to the out-of-plane phenyl C−H wagging and vinylene C−H wagging, respectively. The intensities of these two peaks are expected to be higher when phenyl-vinyl planes are aligned parallel to the substrate surface and upon thermal annealing the film, the peaks at 1033 and 1207 cm^−1^ become little weaker than un-annealed MEH-PPV film because phenyl rings become more parallel to the substrate^33^. The bands at 1033 and 1260 cm^−1^ are attributed to aryl alkyl ether (C−O−C) symmetric and asymmetric stretching, respectively. Phenyl-oxygen stretching can be observed at 1207 cm^−1^. The bands at 1376 and 1458 cm^−1^ exhibit symmetric and asymmetric C–H bending, respectively, in the CH_2_ group. The two sharp bands at 2853 and 2954 cm^−1^ are assigned to asymmetric C−H stretching in the CH_2_ and CH_3_ group, respectively.

The FT-IR spectra of the developed ZnO thin films showed various characteristic peaks. The appearance of a weak absorption peak at around 485 cm^−1^ represents the existence of Zn−O stretching mode. The peaks at 1064 and 1416 cm^−1^ are attributed to the aromatic C = C stretching mode and C−O stretching frequencies respectively which may be due to the traces of surfactant. The broad band around 3400 cm^−1^ is assigned to the existence of the hydroxyl group on the surface of the sample and the weak absorption at 2500 cm^−1^ is due to the existence of CO_2_ molecules. The bands at 2800–3000 cm^−1^ are due to C−H stretching frequencies.

The FT-IR spectra of PEDOT:PSS film have peaks at 836 and 947 cm^−1^, which correspond to the vibrations of the S–C bonds in the polymerized PEDOT chain. The peaks at 1073 and 1133 cm^−1^ are assigned to the stretching of the C−O−C bonds in the ethylene dioxy group. The peaks at 1220, 1333, 1460, and 1520 cm^−1^ may be attributed to the stretching of the thiophene ring. The presence of the peaks at 2883 and 2930 cm^−1^ are due to alkyl C−H stretching vibrations. The peaks at 1646 and 3357 cm^−1^ correspond to the O−H bending and stretching vibrations, respectively.

Functional thin films of different categories of conductive materials have been successfully deposited with excellent morphological, chemical, and electrical characteristics. The film has also been used in device fabrication and a very good device performance has been achieved. The hybrid SAW-EHDA system for functional thin film deposition and pattern fabrication is a promising novel technique with certain advantages of small and uniform atomized particle size, low power consumption, ambient atmospheric conditions, and excellent film characteristics. It is a promising technique proposed to be used for device fabrication such as sensors and FETs.

### Applications

**Figure 7:**
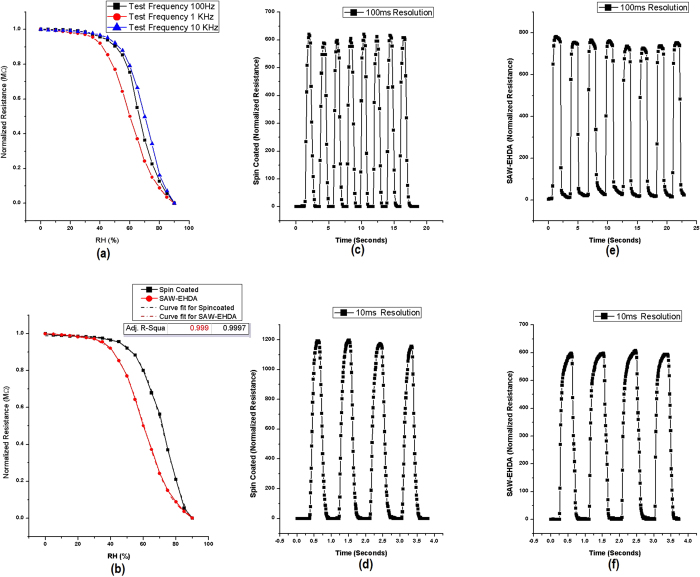
Characteristic curves of the sensors’ response measured at varying humidity levels. (**a**) Response of SAW-EHDA based sensor at different test frequencies for a %RH range of 0%RH to 90%RH, (**b**) Response time of spin coated sensor with a measurement resolution of 100 ms, (**c**) Response time of SAW-EHDA based sample with a measurement resolution of 100 ms, (**d**) Comparison of resistive response of spin coated sample with SAW-EHDA based sample towards a full measurement range of varying humidity levels at 1 KHz test frequency, (**e**) Response time of spin coated sensor with a measurement resolution of 10 ms, (**f**) Response time of SAW-EHDA based sample with a measurement resolution of 10 ms.

The hybrid SAW-EHDA system has been successfully implemented to deposit various conductive functional thin films. The fabricated films exhibit excellent electrical and morphological properties and can be used in various applications. As the phenomenon works on the principle of atomization, the porosity of the film is expected to be higher than that of the same film deposited using spin-coating. This fact was taken under consideration and a humidity sensor based on PEDOT:PSS thin film as the sensing layer was fabricated and characterized. The same IDT used to produce surface acoustic waves was used as the IDT for the fabrication of humidity sensor. Using a piezoelectric substrate and a high frequency IDT for humidity sensing gives additional advantages of fast recovery times of the sensors as explained in our previous work^34^. To compare the response of sensor fabricated using SAW-EHDA, another sensor was also fabricated with the active layer deposited using spin coating. The resistance response of the sensor fabricated through SAW-EHDA was recorded at different test frequencies for varying humidity range. The response of spin coated sample was also recorded at 1 kHz test frequency (best results) and was compared with that of SAW-EHDA sample. The response and recovery time graphs were also recorded using the breath detection system as mentioned in our previous research work^34^. Figure 7 shows the results and comparison of both types of sensors in terms of measurement efficiency and also response and recovery time graphs.

Figure 7a shows the excellent humidity sensing properties of the sensor with active layer fabricated using SAW-EHDA covering a wide measurement range from 0% RH to 90% RH. The comparison between the responses of two sensors towards varying humidity levels for complete range is presented in Fig. 7b. The graph indicates that the curve for spin coated sample lies higher than that of its SAW-EHDA counterpart with spin coated sample having very low sensitivity in the lower RH region. Also, the slope of the spin coated sensor is higher implying that the sensor is not suitable for wide range humidity sensing applications. The response and recovery times were recorded with a resolution of 100 ms and 10 ms for both the sensors. Figure 7c,d show the response and recovery times towards inhaling and exhaling of the spin coated sample with a resolution of 100 ms and 10 ms respectively. Figure 7e,f show the response and recovery times of the SAW-EHDA sample with a resolution of 100 ms and 10 ms respectively. The high resolution graphs indicate that the response time of spin coated sample was around 195 ms (average of four readings) while that of SAW-EHDA sample is 140 ms. The recovery times of spin coated and SAW-EHDA samples are 180 ms and 120 ms respectively. Although there is not a big difference in the operation speeds of the sensors, but it is clear that the SAW-EHDA based samples show better operation speeds as well as sensitivity. One more trend that can be observed from the response time plots is the stability in operation in case of SAW-EHDA based sensor with smooth and stable curves at constant humidity level while sharp transitions between higher and lower humidity levels.

**Figure 8:**
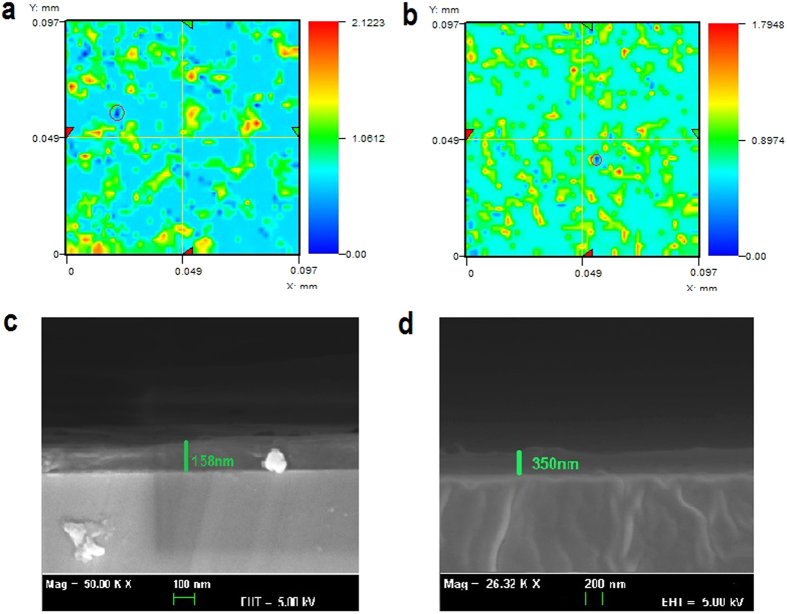
Comparison of film morphologies of the two sensors fabricated using SAW-EHDA deposition and spin coating. (**a**) 2D surface profile of SAW-EHDA based film (**b**) 2D surface profile of spin coated film (**c**) Cross sectional SEM image of SAW-EHDA based film (**d**) Cross sectional SEM image of spin coated film.

The cross sectional SEM and 2D surface profile images of both the spin coated PEDOT:PSS active layer and SAW-EHDA active layer are presented in Fig. 8 showing that the film thickness of spin coated film is 350 nm while that of SAW-EHDA film is 158 nm that is considerably lower than that of spin coated sample. The 2D surface profile images of SAW-EHDA based film and spin coated film are presented in Fig. 8a,b respectively. The results indicate that the number of pores (dark blue spots) present in the spin coated film is much lower (almost half) as compared to the one deposited using SAW-EHDA for the same area of sample. The average pore size in SAW-EHDA based sample is also larger than that of spin coated sample. This means that the porosity of the SAW-EHDA based sample is almost double than that of the spin coated sample.

A comprehensive comparison of a similar sensor fabricated using the same high frequency IDT on piezoelectric substrate with the other humidity sensors previously reported in literature has been presented in a tabular form in our previous research work^34^. The measurable range of the sensor fabricated using SAW-EHDA is even wider than our previous work that is due to the higher film porosity. The response time of SAW-EHDA based sensor is also almost three times faster than our previous work owing to increased film porosity and reduced film thickness. Also, if we compare our sensor’s characteristics with the ones reported in literature fabricated using pure PEDOT:PSS as the sensing layer, the measurable range achieved is 20%RH to 65%RH in one work^35^ and the range achieved in another work is 25%RH to 95%RH^36^. Both measurable ranges are considerably lower as compared to the SAW-EHDA based device. The response and recovery times in both the referred works are also quite slower than ours. The film morphology and the sensors’ response show that the film fabricated using SAW-EHDA is highly suitable for sensing applications.

## Methods

### Synthesis of MEH-PPV ink

MEH-PPV ink (conc. 0.5% wt.) was prepared for this experiment. MEH-PPV powder was dissolved in chloroform and kept for bath sonication for 5 min at 4 °C and then kept on a magnetic stirrer at room temperature for 30 min.

### Synthesis of ZnO ink

ZnO ink (conc. 5% wt.) was prepared for the SAW-ED experiment. ZnO powder was dispersed in 99.9% ethanol and then sonicated for 10 min using ultrasonic probe sonicator. 0.01% Triton X-100 was added as surfactant to reduce the surface tension and enhance dispersion of particles that facilitates the spray and remove agglomeration of the ink. The solution was then stirred for 4 hours at 70 °C after which the ink was ready to be used.

### Synthesis of PEDOT:PSS ink

PEDOT:PSS (conc. 5% wt.) was prepared by diluting PEDOT:PSS paste into iso-propyl alcohol and ultra-sonicated for 30 min. Ink was then placed on a magnetic stirrer at room temperature for 3 hours. The physical properties of the inks are given in supplementary Table 2.

### Sensor Fabrication and Characterization

The sensor was fabricated by replacing the target substrate in the experimental setup with another high frequency IDT on LiNbO_3_ as the sensor electrode and focusing the collimator opening at the active portion of the IDT. PEDOT:PSS was atomized using the same parameters as mentioned in Table 1 and a thin film was deposited as the active layer of the humidity sensor. To get a thin and porous film suitable for sensing applications, the deposition time was reduced from 30 minutes to 5 minutes. The active layer of the other sensor fabricated for comparison was deposited using simple spin coating by masking the connection portions at the electrodes and dropping the same PEDOT:PSS ink using micro-pipit at the active area of the IDT and then spin coating at 2500 rpm.

The sensors were characterized by placing the fabricated sensor in a sealed chamber along with a reference humidity sensor and then controlling the level of humidity inside the chamber by varying the water vapor contents and the Nitrogen gas flow rate through controlled valves. The humidity level readings and the sensor’s real time resistance and impedance were logged in to the computer using USB interface with time stamps. The details on our characterization setup and the schematic diagram are presented in our previous research work^34^.

### Equipment

The characterization of the SAW-EHDA deposited MEH-PPV, PEDOT: PSS, and ZnO thin films was carried out using various techniques. An Olympus BX51M optical microscope equipped with image analysis capabilities was used for the optical micrographs. A state-of-the-art, nondestructive, thin film thickness measurement system, K-MAC ST4000-DLX, based on the principle of constructive and destructive interference in the spectrum of white light incident on the surface of the film was used for the measurement of film thickness. A JSM-6700F field-emission scanning electron microscope (FE-SEM) was used to observe the film’s morphology, conformity and cross sectional thickness. The quantitative analysis of surface morphology was carried out using a NanoView high accuracy 3D nano non-contact surface profiler. A micro Raman spectrometer system under the model of LabRAM HR EV was used with a 514 nm wavelength laser as an excitation source for the Raman analysis. The functional groups present in the film were investigated using Fourier transform infrared spectroscopy (FT-IR) analyzer, BrukerIFS66/S-Germany. The UV characterization was performed using a Shimadzu UV-3150 UV/VIS/NIR spectrophotometer. Agilent B1500A semiconductor device analyzer coupled with MST8000C probe station was used for electrical characterization. Four circular silver contacts each with a diameter of 0.5 mm at a separation distance of 3 mm from each other forming a square were deposited on top of the surfaces. Then the I–V data was recorded by connecting the probes of the device analyzer to any two of the contacts one by one for all four sides of the square. Single and double I-V sweeps at different voltages and step sizes were recorded for the samples to ascertain the stability of results and the curves were plotted in real time.

## Additional Information

**How to cite this article**: Choi, K. H. *et al.* Hybrid Surface Acoustic Wave- Electrohydrodynamic Atomization (SAW-EHDA) for the Development of Functional Thin Films. *Sci. Rep.* **5**, 15178; doi: 10.1038/srep15178 (2015).

## Supplementary Material

Supplementary Video S1

Supplementary Video S2

Supplementary Information

## Figures and Tables

**Table 1:** SAW-EHDA experimental parameters used for the deposition of various thin films.

	Parameters	MEH-PPV	PEDOT:PSS	ZnO
1	Nozzle diameter	210 μm	210 μm	210 μm
2	Flow rate	750 μl/h	700 μl/h	700 μl/h
3	Mask diameter	5 mm	3 mm	3 mm
4	Nozzle to IDT distance	5 mm	5 mm	5 mm
5	IDT to mask distance	40 mm	35 mm	30 mm
6	Mask to substrate distance	10 mm	10 mm	10 mm
7	Nozzle (−ve)—applied voltage	−4.07 kV	−4.539 kV	−4. 72 kV
8	Mask (+ve)—applied voltage	1.312 kV	1.50 kV	1.6 kV
9	Substrate (+ve)—applied voltage	3.27 kV	3.35 kV	3.52 kV
10	Applied frequency	19.8 MHz	19.8 MHz	19.8 MHz
11	Power	5 W	5 W	6 W
12	Deposition time	30 min	30 min	30 min

IDT, Interdigitated transducer.
