# Supplementary material for: Hybrid Surface Acoustic Wave- Electrohydrodynamic Atomization (SAW-EHDA) For the Development of Functional Thin Films
Source: Sci Rep. 2015 Oct 19;5:15178. doi: 10.1038/srep15178 (PMC4609910; doi:10.1038/srep15178)
Supplement: Supplementary Information [file srep15178-s3.pdf]

Hybrid Surface Acoustic Wave-Electrohydrodynamic Atomization (SAW-EHDA) For the Development of Functional Thin Films

Kyung Hyun Choi[1,], Hyun Bum Kim[1], Kamran Ali[1], Memoon Sajid[1], Ghayas Uddin Siddiqui[1], Dong Eui Chang[2], Hyung Chan Kim[3], Jeong Beom Ko[4], Hyun Woo Dang[4], and Yang Hoi Doh[4]*

[1]Department of Mechatronics Engineering, Jeju National University, Jeju 690-756, South Korea

[2]Department of Applied Mathematics University of Waterloo, Canada

[3]Korea Institute of Industrial Technology, South Korea

[4]Department of Electronics Engineering, Jeju National University, Jeju 690-756, South Korea

Supplementary Information

* E-mail: amm@jejunu.ac.kr

*Phone: +82-64-754-3713

*Fax: +82-64-752-3174

Table 1│ The comparison of SAW-EHDA with other deposition techniques						
**Materials**	**Deposition Technique**	**Deposition rate or Thickness**	**Electrical results**	**Transmittance**	**Roughness (RMS)**	**Reference**
ZnO	Magnetron sputtering	11 nm/min	4.5 kΩ	90 %	1.48 nm	1,2
	Chemical vapor deposition	20 nm/min	3x10-4 Ωcm	83 %	1.31 nm	3,4
	Electrospray	600 nm	64 Ωcm	80-88 %	69.3 nm	5,6
	SAW-EHDA	54 nm/min	959 m Ωcm, 1.04 s/cm	90 %	9.31 nm	Present work
MEHPPV	Electrospray	168 nm	14 kΩ	80 %	11.5 nm	7
	Spin Coat	140 nm	1x10-5 Ωcm	80 %	0.98 nm	8–10
	SAW-EHDA	67 nm/min	1.125 Ωcm, 0.889 s/cm	85 %	16.18 nm	Present work
PEDOT:PSS	Spray coating	180 nm	49.6 m Ωcm	82 %	11 nm	11,12
	Spin coating	93 nm	443 s/cm	89 %	1.0 nm	13
	Spray, spray +brush deposition	635 nm	98.87 s/cm	84 %	14 nm	14,15
	SAW-EHDA	70 nm/min	479 mΩcm, 2.09 s/cm	79 %	8.09 nm	Present work

Supplementary Table 1. The comparison of SAW-EHDA with other deposition techniques.

Refrences:

1. Youssef, S., Combette, P., Podlecki, J., Asmar, R. Al & Foucaran, A. Structural and Optical Characterization of ZnO Thin Films Deposited by Reactive rf Magnetron Sputtering. *Cryst. Growth Des.* **9,** 1088–1094 (2009).

2. Damiani, L. R. & Mansano, R. D. Zinc oxide thin films deposited by magnetron sputtering with various oxygen/argon concentrations. *Journal of Physics: Conference Series* **370,** 012019 (2012).

3. Purica, M., Budianu, E., Rusu, E., Danila, M. & Gavrila, R. Optical and structural investigation of ZnO thin films prepared by chemical vapor deposition (CVD). *Thin Solid Films* **403-404,** 485–488 (2002).

4. Li, X. *et al.* Chemical vapor deposition-formed p-type ZnO thin films. *Journal of Vacuum Science & Technology A: Vacuum, Surfaces, and Films* **21,** 1342 (2003).

5. Hwang, K. S., Jeong, J. H., Jeon, Y. S., Jeon, K. O. & Kim, B. H. Electrostatic spray deposited ZnO thin films. *Ceram. Int.* **33,** 505–507 (2007).

6. Muhammad, N. M., Naeem, A. M., Duraisamy, N., Kim, D. S. & Choi, K. H. Fabrication of high quality zinc-oxide layers through electrohydrodynamic atomization. *Thin Solid Films* **520,** 1751–1756 (2012).

7. Choi, K. H., Mustafa, M., Ko, J. B. & Doh, Y. H. Investigation of electrostatic atomization of a conjugated polymer (poly[2-methoxy-5-(2'-ethylhexyloxy)-(p-phenylenevinylene)]) and its film characterization for organic diode applications. *Thin Solid Films* **525,** 40–44 (2012).

8. Yang, Y., Yijian, S., Jie, L. & Tzung-Fang, G. The control of morphology and the morphological dependence of device electrical and optical properties in polymer electronics. Electronic and Optical Properties of Conjugated Molecular Systems in Condensed Phases **661,** 307-354 (2003).

9. Weszka, J, M.M. Szindler, M. & Szczesna, M. S. Influence of solvent on the surface morphology and optoelectronic properties of a spin coated polymer thin films. *J. Achiievements Mater. Manuf. Eng.* **61,** 302–307 (2013).

10. Choi, G. W., Lee, W. S. & Seo, Y. J. Improvements of Electrical and Optical Property of Organic Light Emitting Diode using Chemical Mechanical Polishing Process. International Conference on Planarization/CMP Technology, Dresden, Germany. 1-6 (IEEE, 2007).

11. Zabihi, F., Xie, Y., Gao, S. & Eslamian, M. Morphology, Conductivity and Wetting Characteristics of PEDOT:PSS Thin Films Deposited by Spin and Spray Coating. *Appl. Surf. Sci.* **338,** 163–177 (2015).

12. Duraisamy, N., Muhammad, N. M., Ali, A., Jo, J. & Choi, K. H. Characterization of poly(3,4-ethylenedioxythiophene):poly(styrenesulfonate) thin film deposited through electrohydrodynamic atomization technique. *Mater. Lett.* **83,** 80–83 (2012).

13. Yan, H., Jo, T. & Okuzaki, H. Highly Conductive and Transparent Poly(3,4-ethylenedioxythiophene)/Poly(4-styrenesulfonate) (PEDOT/PSS) Thin Films. *Polymer Journal* **41,** 1028–1029 (2009).

14. McCarthy, J. E., Hanley, C. A., Brennan, L. J., Lambertini, V. G., & Gun'ko, Y. K. Fabrication of highly transparent and conducting PEDOT:PSS films using a formic acid treatment. *J. Mater. Chem. C* **2,** 764-770 (2014).

15. Chilvery, A. K., Batra, A. K., Guggilla, P., Lal, R. B. & Surabhi, R. A Versatile Technique for the Fabrication of PEDOT : PSS Films for Organic Solar Cells. *Energy Sci. Technol.* **4,** 6–11 (2012).

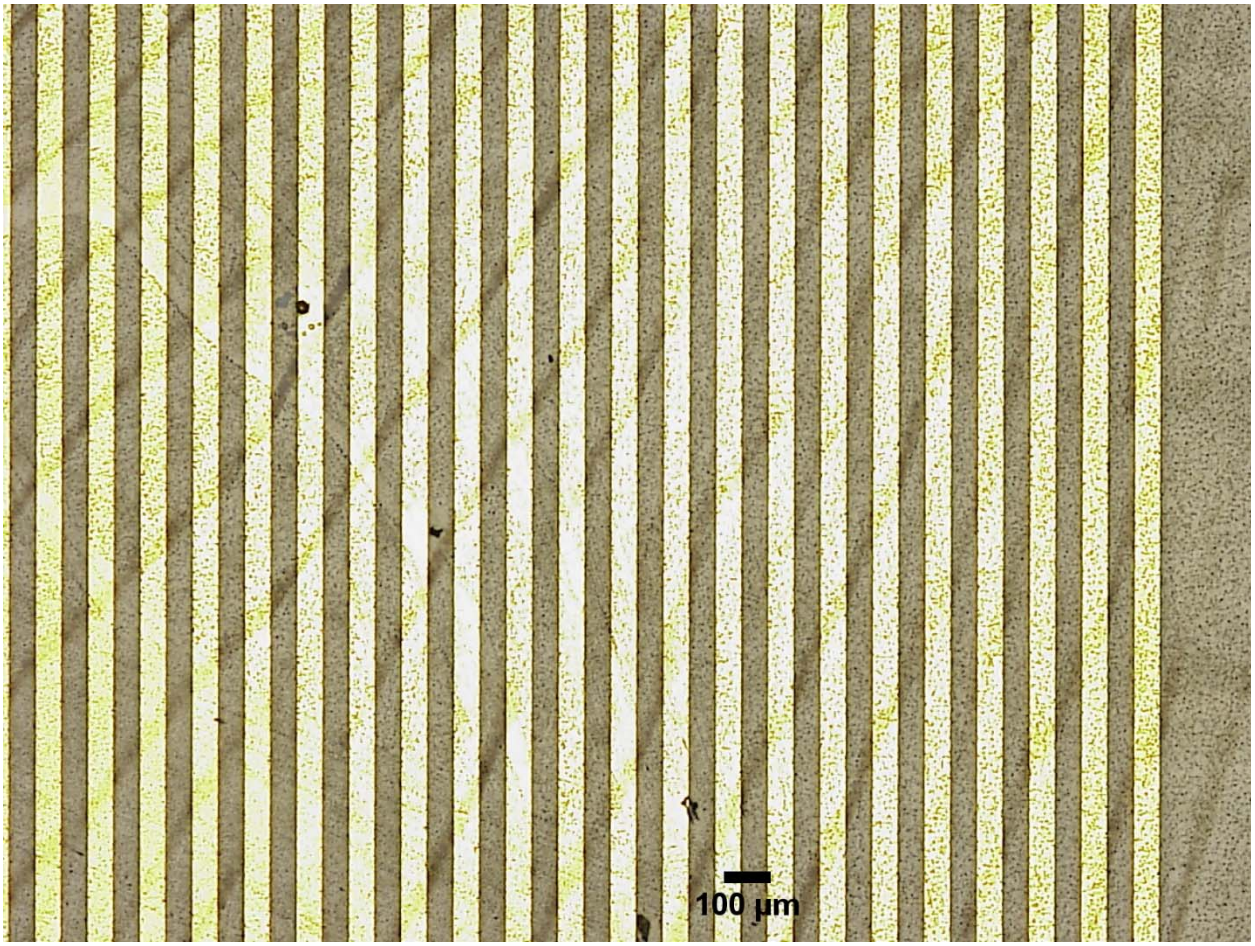

Supplementary Figure 1. The Dimensions of the IDT

An important parameter in atomization process is the atomized particle diameter that can be found using equation-2 [18] which relates the mean particle diameter with the excitation frequency.

$$d_o = (\gamma\varepsilon^2/\rho K^2)^{1/3} \qquad (2)$$

Here γ is the surface tension of the ink, ρ is the density and K is the ink conductivity. The average surface tension in our case is 28mN/m, while the average conductivity is 20uS/m. In

this case of 19.8MHz atomizer, the average theoretical diameter of the droplet comes out to be approximately 0.5µm.

The relationship for jet diameter in EHDA is shown in expression 3. [24]

$$d_{jet} \approx 0.4[Q\epsilon_o \epsilon_r / K]^{1/3} \quad (3)$$

Here Q is the flow rate of the ink and K is the conductivity. The critical voltage that supports the meniscus on the capillary tube can be found using equation 4 [25].

$$V_c = \sqrt{\gamma d / \epsilon_o} \quad (4)$$

Here d is the capillary diameter and γ is the surface tension of the liquid. The droplet size for EHDA can be calculated using equation 5 [24].

$$d_d = (\rho \epsilon_o Q^3 / \gamma K)^{1/6} \approx Q^{0.48} \quad (5)$$

In this equation, ρ is the density of the liquid, Q is the flow rate, γ is the surface tension and K is the conductivity.

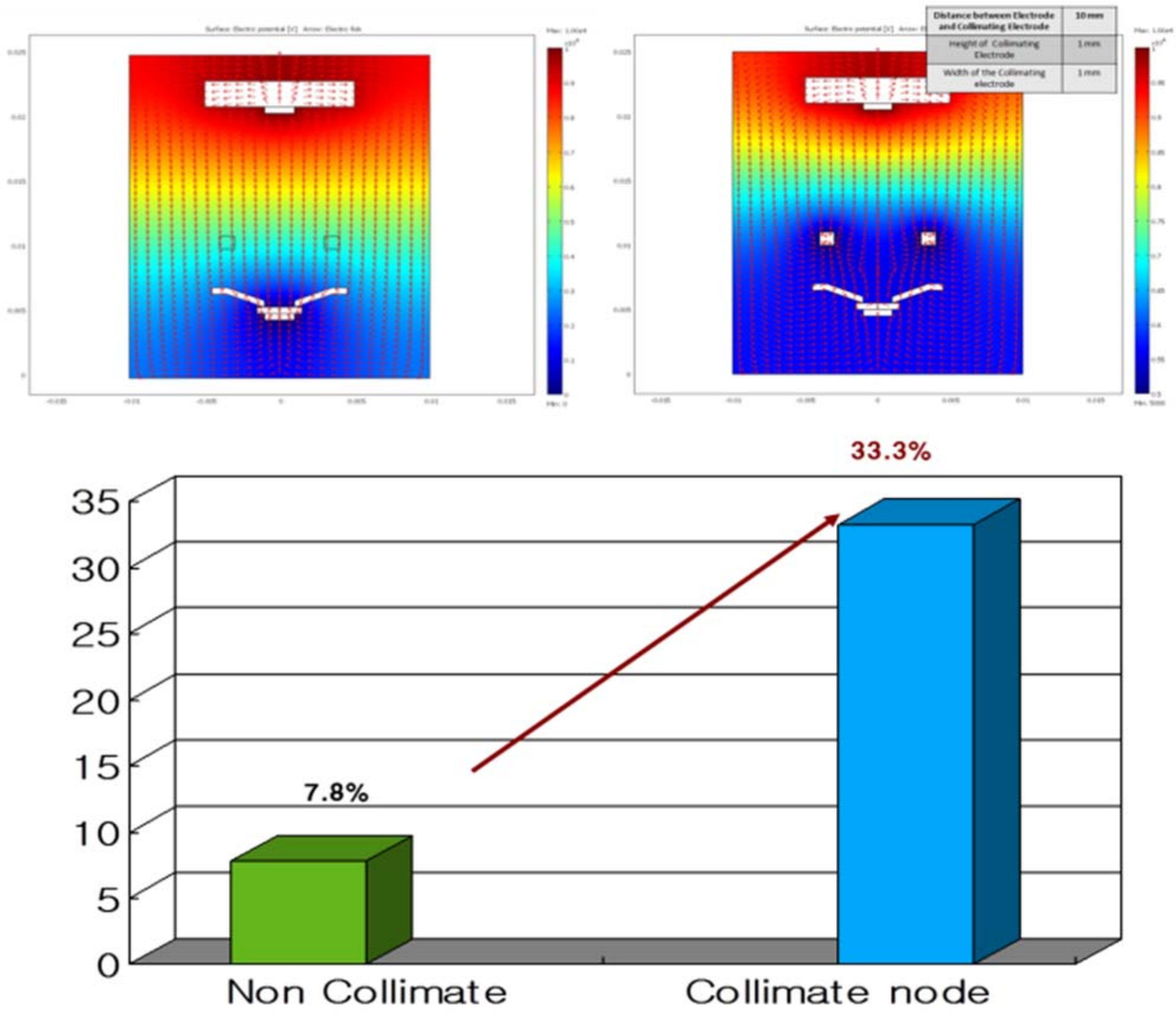

Supplementary Figure 2: COMSOL simulation of electric field concentration with and without collimator defining the droplet collection efficiency of the SAW-EHDA deposition system

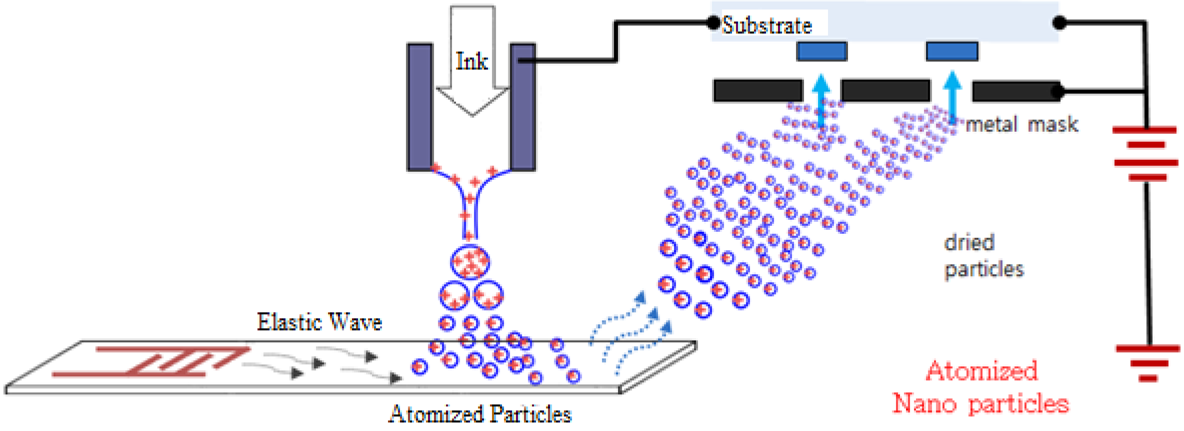

Supplementary Figure 3: Working Principle of the Hybrid SAW-EHDA Deposition System

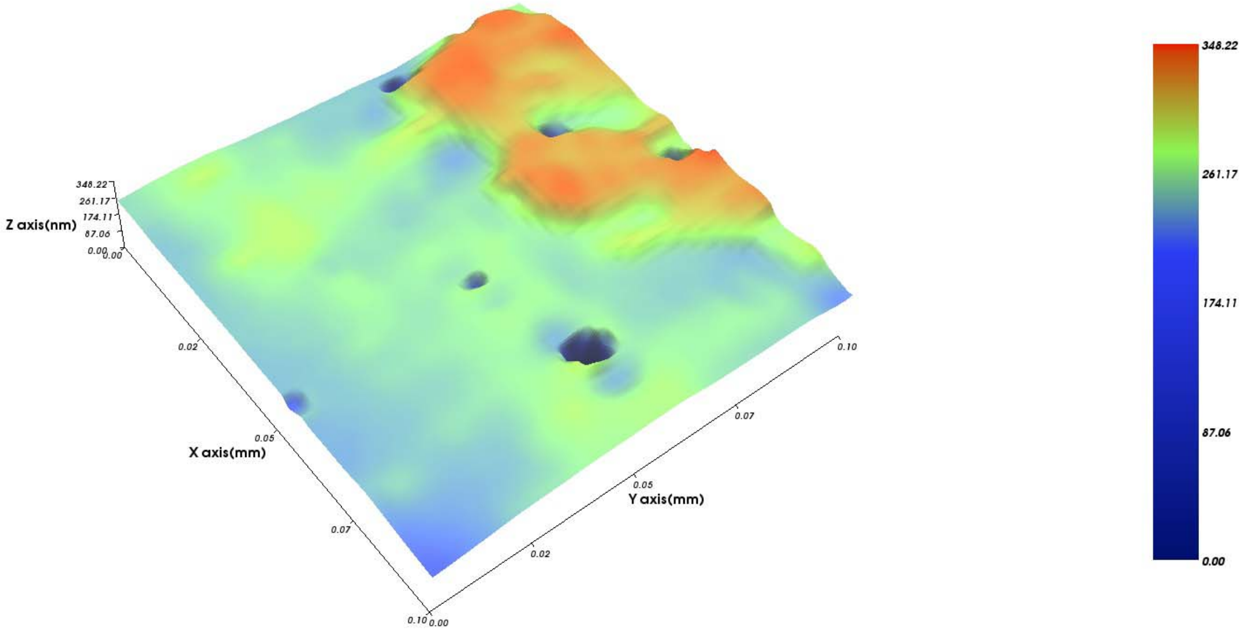

Supplementary Figure 4. 3D surface profile of MEH-PPV thin film. The Ra, Rq, Rt, and Rz of the film were 16.18 nm, 22.81 nm, 317.27 nm, and 217.73 nm respectively.

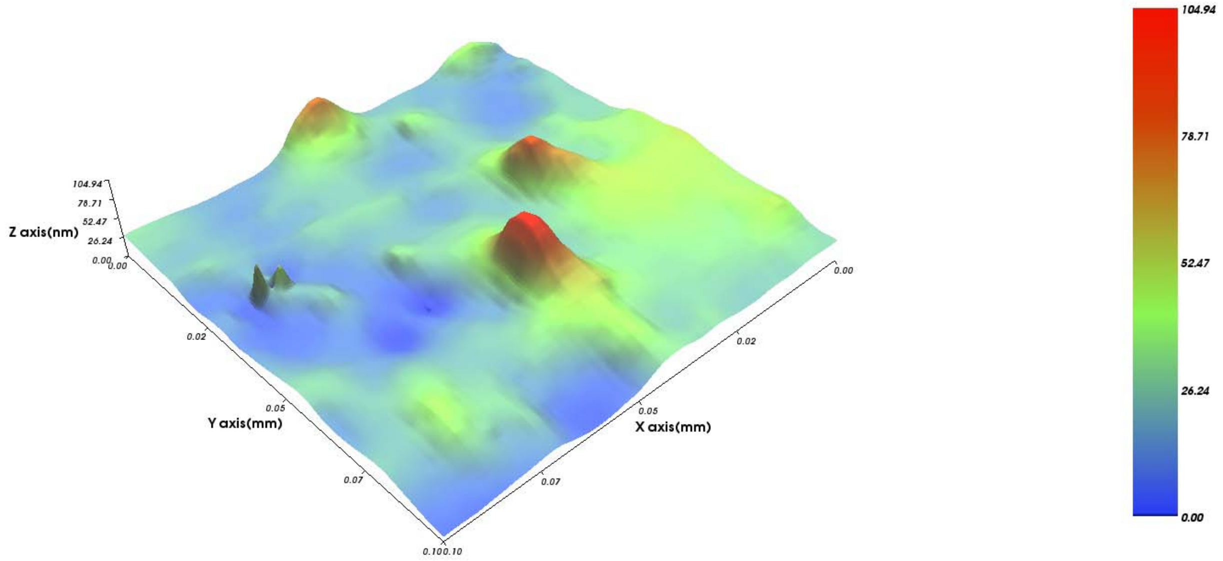

Supplementary Figure 5. 3D surface profile of PEDOT:PSS thin film. The Ra, Rq, Rt, and Rz of the film were 8.09 nm, 11.74 nm, 100.67 nm, and 87.31 nm respectively.

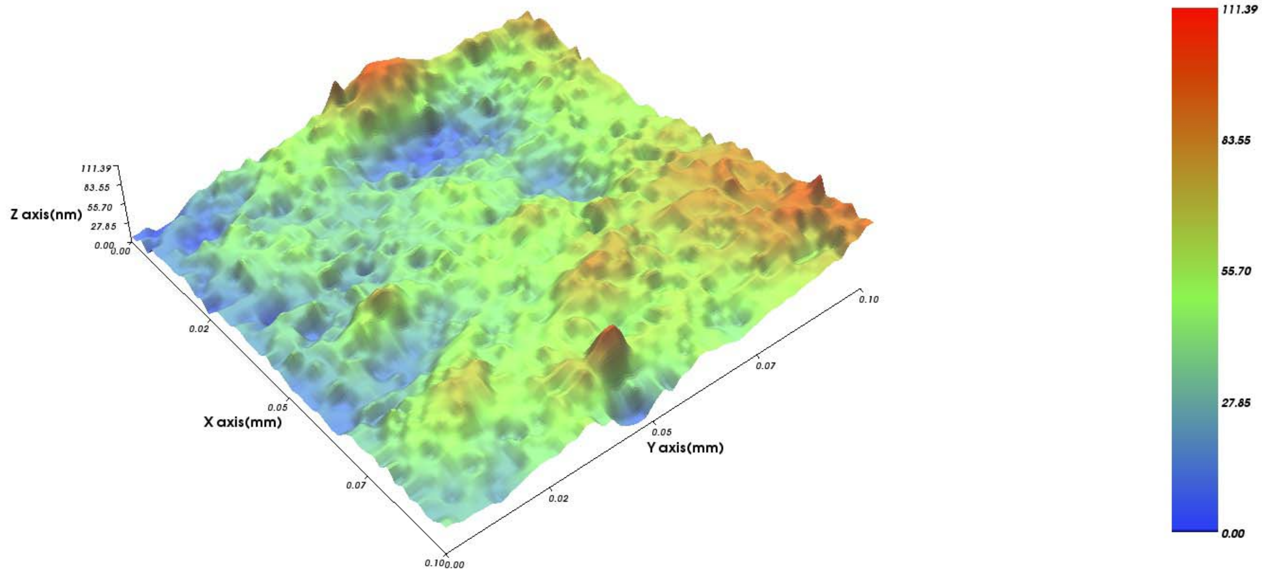

Supplementary Figure 6. 3D surface profile of ZnO thin film. The Ra, Rq, Rt, and Rz of the film were 9.31 nm, 12 .46 nm, 105.20 nm, and 90.36 nm respectively.

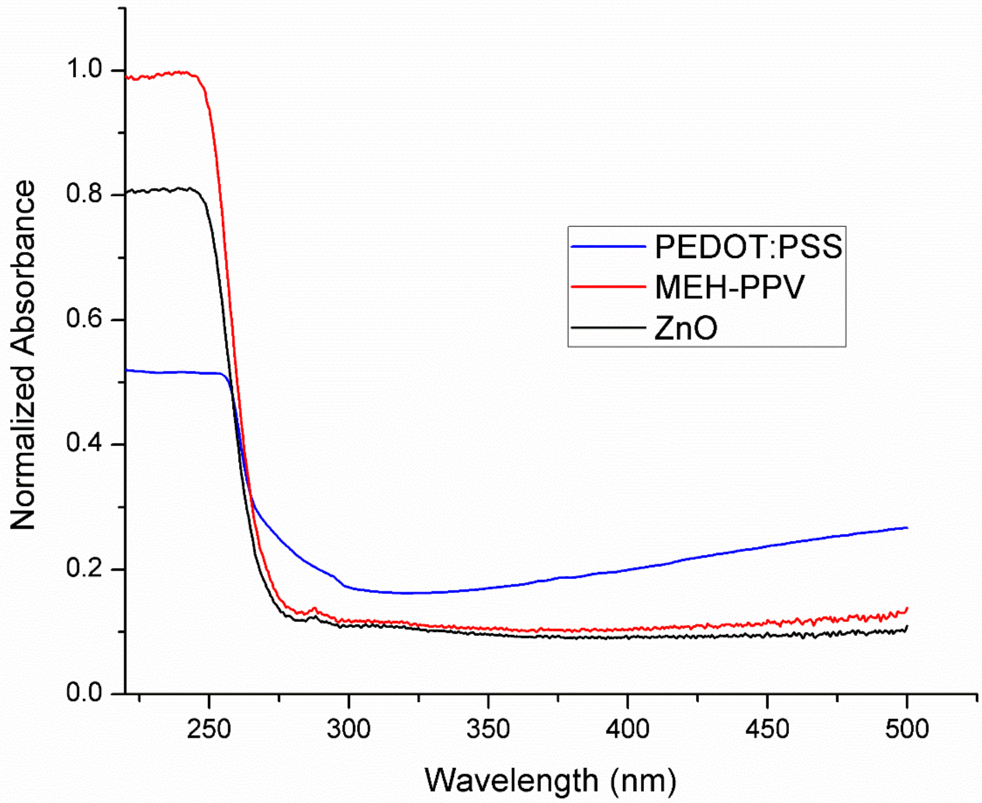

Supplementary Figure 7. The normalized absorbance spectra of ZnO, MEH-PPV, and PEDOT: PSS thin films deposited through SAW-EHDA.

Table 1	Physical properties of the inks used for the deposition of various thin films.			
**S.No**	**Ink (Conc. Wt.%)**	**Viscosity (mPas)**	**Surface tension (mN/m)**	**Conduvtivity (µs/cm)**
1	MEH: PPV (0.5%)	2.52	27.4	2.5
2	ZnO (5%)	7.44	32.3	8.34
3	PEDOT: PSS (5%)	2.9	24.5	4.27

Supplementary Table 2. The Physical properties of the inks used for the disposition of various thin films.

Supplementary Video S1 and S2. The phenomenon of particles atomization by the SAW-EHDA process, ZnO (Video S1) and MEH:PPV (Video S2).
